# Incubation with tau aggregates increases hippocampal circuit excitability and enhances long-term depression in acute mouse hippocampal slices

**DOI:** 10.3389/fncir.2025.1596989

**Published:** 2025-09-19

**Authors:** Alice Wang, Abbie Richardson, Isabelle Emmett, Daniel Friedmann, Saskia Bakker, Magnus Richardson, Emily Hill, Mark Wall

**Affiliations:** School of Life Sciences, University of Warwick, Coventry, United Kingdom

**Keywords:** electrophyisology, long term potentiation (LTP), whole cell patch clamp, tauopathies, synaptic plasiticity

## Abstract

Aggregation of the protein tau is a key pathological hallmark of tauopathies such as Alzheimer’s Disease. Tau dissociates from microtubules and diffuses from the axon into the soma-dendritic compartment, where it aggregates firstly into oligomers and ultimately into neurofibrillary tangles. There is gathering evidence that it is the soluble tau aggregates that are the major active species and that their effects on neuronal electrophysiological properties, synaptic transmission and plasticity could contribute to early cognitive decline. Here we have investigated the effects of incubating acute mouse hippocampal slices with recombinant tau aggregates. We observed interictal events and an increase in excitability of CA3 pyramidal cells. Tau aggregates had little effect on basal synaptic transmission but antagonism of GABA_A_ receptors revealed significant effects of tau aggregates, enhancing the firing of population spikes and the occurrence of bursts following fEPSPs. Tau aggregates produced a concentration-dependent impairment of long-term potentiation (LTP), which could not be overcome by repeated LTP induction stimuli, demonstrating the effects were not just through an elevation of LTP threshold. In contrast to the impairment of LTP, tau aggregates increased G1-mGluR-dependent LTD. Thus, tau aggregates increase hippocampal circuit excitability and shift synaptic plasticity towards depression.

## Introduction

Tau is a soluble protein, primarily found in neurons, which plays a major role in maintaining neuronal morphology by binding to microtubules (reviewed in Tracy and Gan, 2018; Waheed et al., 2023; Parra Bravo et al., 2024). Tau also assists with the axonal transport of vesicular cargo through interaction with the microtubule-associated motor proteins kinesin and dynein (reviewed in Utton et al., 2005; Magnani et al., 2007). Tauopathies are a pathologically diverse collection of neurological conditions, where dysregulated tau induces neural dysfunction and degeneration (reviewed in Sillen et al., 2007; Sinsky et al., 2021; Devi, 2023). The most prevalent tauopathy is Alzheimer’s disease (AD). In AD, tau is hyperphosphorylated, resulting in its dissociation from microtubules (reviewed in Rawat et al., 2022). This tau can then misfold and polymerise, first into soluble tau aggregates, then into paired helical filaments, and finally into neurofibrillary tangles (NFTs, reviewed in Avila, 2010; Rawat et al., 2022; Devi, 2023). There is increasing evidence that it is the soluble forms of tau that are active, with the toxic effects of tau oligomers observed in the absence of NFT pathology. For example, introduction of tau oligomers into the brain of wildtype rodents induces synaptic, mitochondrial and memory dysfunction (Fá et al., 2016; Ondrejcak et al., 2018). In tau overexpression models, there is neuronal loss and synaptic dysfunction without NFT pathology (Lee et al., 2001; Wittmann et al., 2001; Tanemura et al., 2002; Tatebaysashi et al., 2002; Andorfer et al., 2003; Spires et al., 2006; Yoshiyama et al., 2007; Cowan et al., 2010).

In previous studies, we have investigated the effects of tau oligomers on neuronal function by introducing nanomolar concentrations of oligomeric full-length tau into mouse hippocampal and neocortical pyramidal cells (Hill et al., 2019). Tau oligomers induced distinct changes in electrophysiology and synaptic functions, which were not observed with equivalent concentrations of monomeric tau (Hill et al., 2019).

In this study we have used slice-incubation experiments to investigate the effects of tau aggregates on hippocampal synapses and on circuit excitability. We found that the incubation of hippocampal slices increased the excitability of CA3 pyramidal cells with little effect on CA1 pyramidal cells. Incubation with tau aggregates had little effect on basal synaptic transmission under control conditions but, following the block of GABAergic inhibition, tau aggregates were pro-epileptic. Tau aggregates produced a concentration-dependent impairment of long-term potentiation - an effect that was not just the result of an increase in the threshold for LTP but also an inhibition of downstream mechanisms. In contrast, to the impairment of LTP, tau aggregates produced a small but significant increase in mGluR-dependent long-term depression, suggesting that tau aggregates can shift the dynamic range of synapses towards depression.

## Methods

### Acute hippocampal slice preparation

C57BL/6 (3–5 weeks old, male and female) mice were kept in standard (group) housing with littermates maintained on a 12:12 (light–dark) cycle. All animal care and experimental procedures were reviewed and approved by the institutional animal welfare and ethical review body (AWERB) at the University of Warwick. Hippocampal slices were produced at times between ZT1 and 2. Mice were sacrificed by cervical dislocation and decapitated in accordance with the UK Animals Scientific Procedures Act (1986). Following decapitation, the brain was rapidly isolated and submerged in high magnesium, low calcium artificial cerebral spinal fluid (aCSF) as follows (in mM): 127 NaCl, 1.9 KCl, 8 MgCl_2_, 0.5 CaCl_2_, 1.2 KH_2_PO_4_, 26 NaHCO_3_, 10 D-glucose (pH 7.4 when bubbled with 95% O_2_ and 5% CO_2_, ~300 mOSM). The brain was cut in half down the midline and sliced (parasagittal 350–400 μm) in (2–4°C) high magnesium, low calcium aCSF using a Microm HM Microslicer. Slices were allowed to recover in standard aCSF (1 mM MgCl_2_, 2 mM CaCl_2_) at 33°C for at least 1 h before recording.

### Whole-cell patch-clamp recording from CA1 and CA3 hippocampal pyramidal cells

A 350 μM slice was transferred to the recording chamber, submerged and perfused (2–3 mL/min^−1^) with aCSF at 30°C. Slices were visualized using IR-DIC optics with an Olympus BX151W microscope (Scientifica) and a CCD camera (Hitachi). Whole-cell current-clamp recordings were made from pyramidal cells in area CA1 and CA3 of the hippocampus using patch pipettes (5–10 MΩ) manufactured from thick-walled glass (Harvard Apparatus). Pyramidal cells were identified by their position in the slice, morphology (from fluorescence imaging) and characteristics of the standard current–voltage relationship. Voltage recordings were made using an Axon Multiclamp 700B amplifier (Molecular Devices) and digitized at 20 kHz. Data acquisition and analysis were performed using pClamp 10 (Molecular Devices). The bridge balance was monitored throughout the experiments and any recordings where it changed by > 20% were discarded. Intracellular solution composition: 135 mM potassium gluconate, 7 mM NaCl, 10 mM HEPES, 0.5 mM EGTA, 10 mM phosphocreatine, 2 mM MgATP and 0.3 mM NaGTP (293 mOsm, pH 7.2).

### Stimulation protocols

To extract the electrophysiological properties of recorded neurons, step and naturalistic, fluctuating currents were injected at 10-min intervals for the duration of the recordings (as in Hill et al., 2019; Hill et al., 2021a; Hill et al., 2021b).

### Standard IV protocol

The standard current–voltage relationship was constructed by injecting step currents from −200 pA incrementing by either 50 or 100 pA (1 s) until a regular firing pattern was induced. A plot of step current against voltage response around the resting potential was used to measure the input resistance (gradient of the fitted line).

### Naturalistic current protocol

A current wave form, designed to induce naturalistic, fluctuating voltages, was constructed using the summed numerical output of two Ornstein–Uhlenbeck processes (Uhlenbeck and Ornstein, 1930) with time constants τfast = 3 ms and τslow = 10 ms. This current wave form, which partly mimics the background post-synaptic activity resulting from activation of AMPA and GABA_A_ receptor channels, was injected into cells and the resulting fluctuating voltage recorded. The firing rate was measured from voltage traces evoked by injecting a current wave form of the same gain for all recordings (firing rate ∼2–3 Hz). Action potentials were detected by a manually set threshold and the interval between action potentials measured to give the instantaneous frequency.

### Production of tau aggregates

Aliquots of frozen (−80°C) tau pre-formed fibrils (PFFs, rPeptide, TF-1001-1) were thawed and diluted in either aCSF or whole-cell patch-clamp intracellular solution to make final concentrations of 44, 133 or 444 nM tau aggregates (calculated using the monomeric molecular weight). The tau PFF solutions were ultrasonicated in a Grant ultrasonic XUBAI bath for 15 min (50–60 Hz), as in Polinski et al. (2018), to break the PFFs into soluble aggregates (as per Hill et al., 2021a).

### Analysis of tau aggregates (transmission electron microscopy)

Formvar/carbon-coated 300-mesh copper grids (#S162, Agar Scientific) were glow-discharged using the ELMO system from Cordouan Technologies. Tau preparations (preformed fibrils and fibrils that had been sonicated for 15 min) were pipetted (5 μL) onto the grid and allowed to bind for one minute. Excess samples were removed with a strip of filter paper, and 5 μL of 2% uranyl acetate added for one minute. After removing the excess stain with a strip of filter paper, the grids were imaged using a JEOL-2100F transmission electron microscope.

### Single molecule mass photometry (SMMP)

SMMP measurements were done using a TwoMP mass photometer (Refeyn) where it is possible to image molecules up to 1.5 MDa. Such an approach has previously been used to investigate the properties of other protein aggregates. For example, Awasthi et al. (2023) used mass photometry to investigate the properties of alpha synuclein oligomers in solution. The machine was firstly calibrated as per the manufacturer’s protocol using proteins of known molecular mass: BSA (66 kDa), apoferritin (480 kDa) and thyroglobulin (670 kDa). All measurements involved a total volume of 20 μL in the measuring well: 15–18 μL of measuring buffer (PBS) was first added to the clean wells before protein was added. SMMP measurement of buffer alone verified that the buffer did not contain any particles generating optical contrast. Between 2–5 μL of the samples were added to the buffer at concentrations of 0.1 to 1 mg/mL measurements were carried out in triplicate, with the counts binned and then fitted with gaussian distributions to give a mean ± standard deviation of the protein mass.

### Incubation of acute hippocampal brain slices in soluble recombinant tau

The tau-aCSF solutions were transferred to custom-made incubation chambers, as in Brown et al. (2023), and bubbled with 95% oxygen/ 5% carbon dioxide. Slices were added to the chambers and incubated in varying dilutions of the tau aggregates (44, 133 or 444 nM), or in aCSF (control) for 1 h prior to transfer to the recording chamber. This duration for incubation was chosen, as in our previous experiments using CSF-tau from human patients had robust effects within this time frame (Brown et al., 2023). It has also been reported that exogeneous tau can be detected in neurons after ~ 20 min of exposure (Fá et al., 2016) and thus 1 h gives adequate time for tau aggregates to be internalised.

### Extracellular recording of synaptic transmission and plasticity

Following incubation, single parasagittal slices (400 μm) were transferred into a submerged recording chamber and perfused with standard aCSF (composition as detailed above) maintained at 30–32°C, perfused with 95% oxygen/ 5% carbon dioxide at a 4–6 mL/min flow rate. Field excitatory postsynaptic potentials (fEPSP) were recorded by placing an aCSF-filled microelectrode on the surface of stratum radiatum in the CA1 region of the hippocampus. fEPSPs were evoked with a bipolar concentric stimulating electrode (FHC) stimulating the Schaffer collateral–commissural pathway which was controlled by an isolated pulse stimulator (Digitimer, Model DS3). fEPSPs were evoked every 30 s (0.3 Hz). fEPSPs were defined as epileptiform if they were contaminated with population spikes and bursts following the initial peak. Stimulus input/output curves were generated using stimulus strengths of 2, 5, 10–100 μA (200 μs duration). The baseline for plasticity experiments was set to 40–50% of the maximum input/output response. Paired-pulse facilitation was recorded for an interval range of 50–500 ms (with 5 repeats for each interval). Following paired-pulse stimulation, a 20 min baseline was recorded proceeding plasticity induction. Long term potentiation (LTP) was induced by high-frequency stimulation (100 Hz, 1 s) and fEPSPs were recorded for at least one hour. Short term potentiation (STP) was measured for the first 5 min following induction and long-term potentiation (LTP) was measured for the 50–60 min after induction. For the saturation of LTP experiments the protocol from Kiyama et al. (1998) was used: after a 20-min baseline LTP was induced with HFS (100 Hz, 1 s). Following LTP induction, a HFS stimulation was applied every 15 min until the slope of the fEPSP no longer increased. At this point the level of LTP was taken as saturated.

For group 1 metabotropic glutamate receptor-dependent long-term depression (G1-mGluR-LTD) the CA3 region of slices was removed and fEPSPs were recorded in picrotoxin (50 μM) and L689,560 (5 μM) to block GABA_A_ and NMDA receptors, respectively (Palmer et al., 1997; Gladding et al., 2009; Wu et al., 2024; Privitera et al., 2019; Wall et al., 2018). G1-mGluR-LTD was evoked with DHPG (100 μM), a group 1 mGluR agonist (applied for 10 min) and was recorded for at least 1 h after washing of the DHPG (Wall et al., 2018; Privitera et al., 2019). Short-term depression (STD) was defined as the peak depression during DHPG application and long-term depression (LTD) was measured for the time 50–60 min after DHPG wash.

### Drugs and substances

Picrotoxin (PTX; Sigma Aldritch), trans-2-carboxy-5,7–170 dichloro-4-phenylaminocarbonylamino-1,2,3,4-tetrahydroquionoline (L689,560; Tocris Bioscience: https://www.tocris.com) and dihydroxyphenylglycine (DHPG, Hello Bio) were made up as stock solutions (20 mM, 5 mM and 5 mM, respectively) in either distilled water or dimethyl sulfoxide (DMSO) and then diluted in aCSF on the day of use. The concentration of DMSO in solutions did not exceed 0.1%.

### Statistical analysis

Graphpad Prism was used to conduct all statistical analyses and details are provided in figure legends. Non-parametric Kruskal-Wallis analysis of variance (ANOVA) followed by Dunn’s test for multiple comparisons and Mann–Whitney signed rank statistical tests were used due to sample sizes *n* < 20. Where two-way ANOVA’s were performed, Tukey’s multiple comparisons test was used. Significance was set at *p* < 0.05 for all statistical tests. All data is represented as the mean and standard error of the mean (SEM).

## Results

### Sonication breaks tau preformed fibrils breaks into smaller aggregates

**Figure 1:**
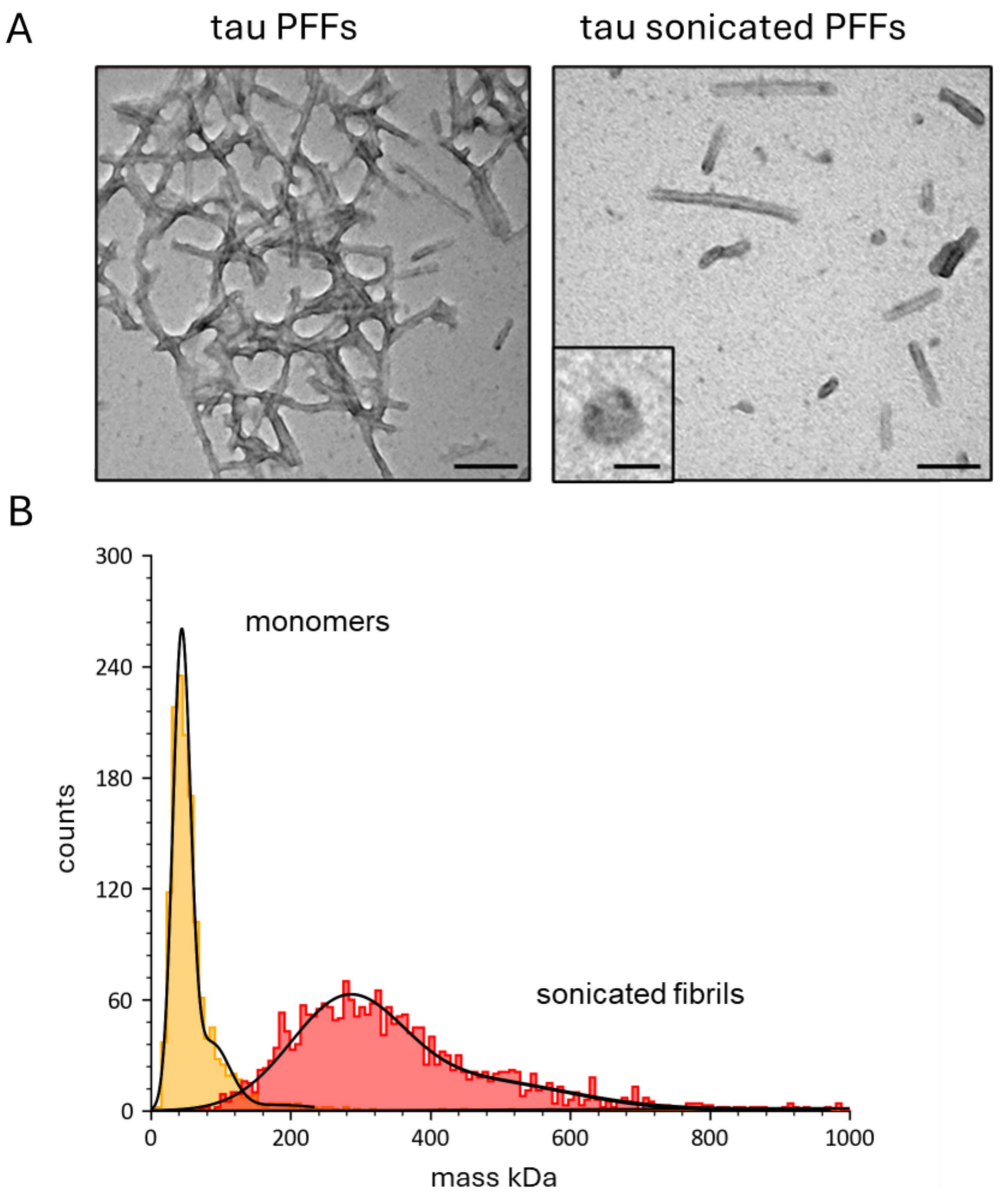
Characterisation of the tau aggregates used for slice incubation. **(A)** Representative negative-stain transmission electron microscopy (TEM) micrographs of tau preformed fibrils before and after sonication (scale bars 100 nm). Inset, a single annular structure at higher magnification (scale bar 20 nm). **(B)** Single-molecule mass photometry (SMMP) measurements of tau species. Graph plotting particle counts against mass (in kDa) for monomers (red) and sonicated preformed fibrils (yellow). The data for each tau species is fitted with single gaussian to give a mean mass (± SD) for the monomers of 50 ± 30 kDa and for the sonicated fibrils of 315 ± 151 kDa.

Recombinant human tau preformed fibrils were purchased from Stratech (PFFs, rPeptide, TF-1001-1). To confirm that the tau species were PFFs, negative stain transmission electron microscopy (TEM) was used to visualise their structure (Figure 1A). The PFFs were broken down with sonication (as in Polinski et al., 2018; Veys et al., 2021) for 15 min (at 50–60 kHz) to produce smaller aggregates to use for slice incubation. TEM confirmed that the fibrils had been broken down into smaller aggregates, with some having a granular structure as previously described for aggregates produced from monomers (Figure 1A, right panel inset as in Hill et al., 2023). The distribution of the particle masses was determined using single molecule mass photometry (SMMP). Tau monomers had a mass of ~ 48 kD and the sonicated fibrils had a mass of ~ 250 kD (Figure 1B) so consisted of ~ 5 monomers.

### Tau aggregates have little effect on the amplitude of basal synaptic transmission but increase paired pulse facilitation and induced interictal events

We investigated if incubation with recombinant tau aggregates changes the properties of hippocampal synaptic transmission, synaptic plasticity and neuronal excitability. We used the same approach as in Brown et al. (2023) and incubated the slices (for 1 h) in small volume chambers (~ 1–2 mL) which were bubbled with O_2_/CO_2_. Slices were incubated in a range of tau aggregate concentrations (44, 133 and 444 nM) as it was unclear at what concentration the tau aggregates would have effects. In our previous experiments, we found that concentrations of tau aggregates between 133–444 nM were active, when introduced into pyramidal neurons during whole cell patch clamp recording (Hill et al., 2019). As a control, slices were incubated in aCSF alone.

**Figure 2:**
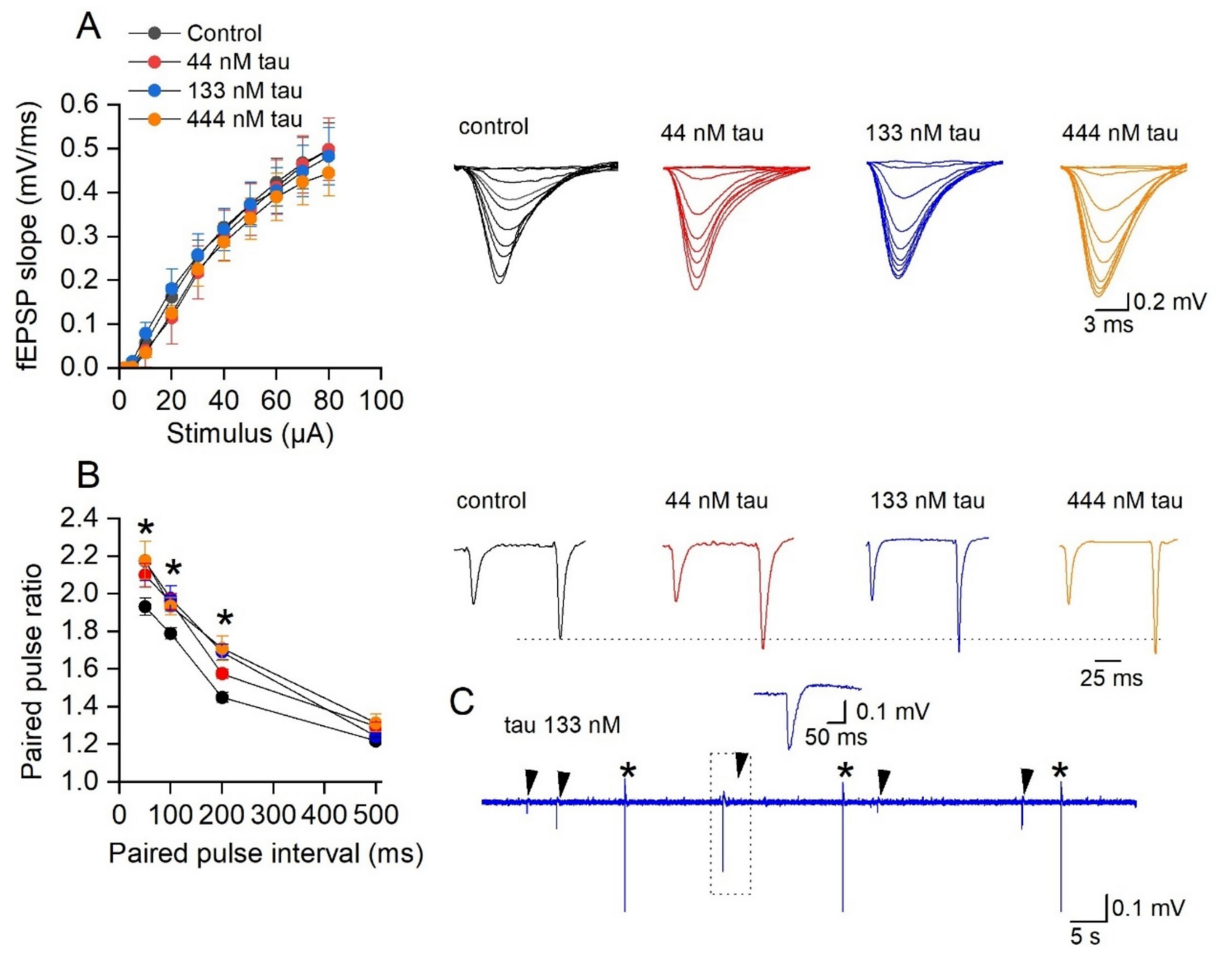
Incubation with soluble recombinant tau aggregates has little effect on synaptic transmission to CA1 pyramidal cells. **(A)** Mean fEPSP slope plotted against stimulus strength for control (*n* = 20 slices), 44 nM (*n* = 8 slices), 133 nM (*n* = *7* slices), and 444 nM tau aggregates (*n* = 6 slices). There was no significant difference produced by incubation in the different concentrations of tau aggregates (Two-way ANOVA). Inset, representative fEPSP waveforms at stimulus strengths of (2–80 μA) for the four conditions. **(B)** Mean paired-pulse ratio plotted against paired pulse interval for control (*n* = *16* slices), 44 nM (*n* = *8* slices), 133 nM (*n* = *10* slices), and 444 nM tau aggregates (*n* = *10* slices). There is a significant difference in the paired pulse ratios across the treatments (Two-way ANOVA, *F* (3, 160) = 197, *p* < 0.0001). Tukey’s multiple comparisons test confirmed the significance (as indicted on the figure). Inset, representative average fEPSP waveforms at a paired pulse interval of 100 ms for the four conditions. The traces are normalised, so the amplitude of the first EPSP is the same as control, illustrating the increase in PPF. **(C)** Examples of spontaneous events (arrows) observed following 133 nM tau incubation between fEPSP stimulations (*). Inset, event outlined in dotted box to show waveform of the spontaneous event.

Firstly, we investigated whether incubation in tau aggregates alters basal synaptic transmission in the CA1 region of the hippocampus. There was no significant change in either the fEPSP waveforms or the stimulus input/output curves for any of the concentrations of tau aggregates vs. control (Figure 2A). However, at the higher concentrations (133 and 444 nM) the tau aggregates significantly increased paired pulse facilitation at some of the intervals (Figure 2B) suggesting effects on transmitter release from presynaptic neurons. Incubation in the tau aggregates (observed with both 133 and 444 nM) also induced frequent spontaneous events that could be observed between fEPSP stimulations (Figure 2C, arrows). The occurrence of these events suggests that the tau aggregates maybe increasing the excitability of the hippocampal network.

### Tau aggregates preferentially affect CA3 pyramidal cells

**Figure 3:**
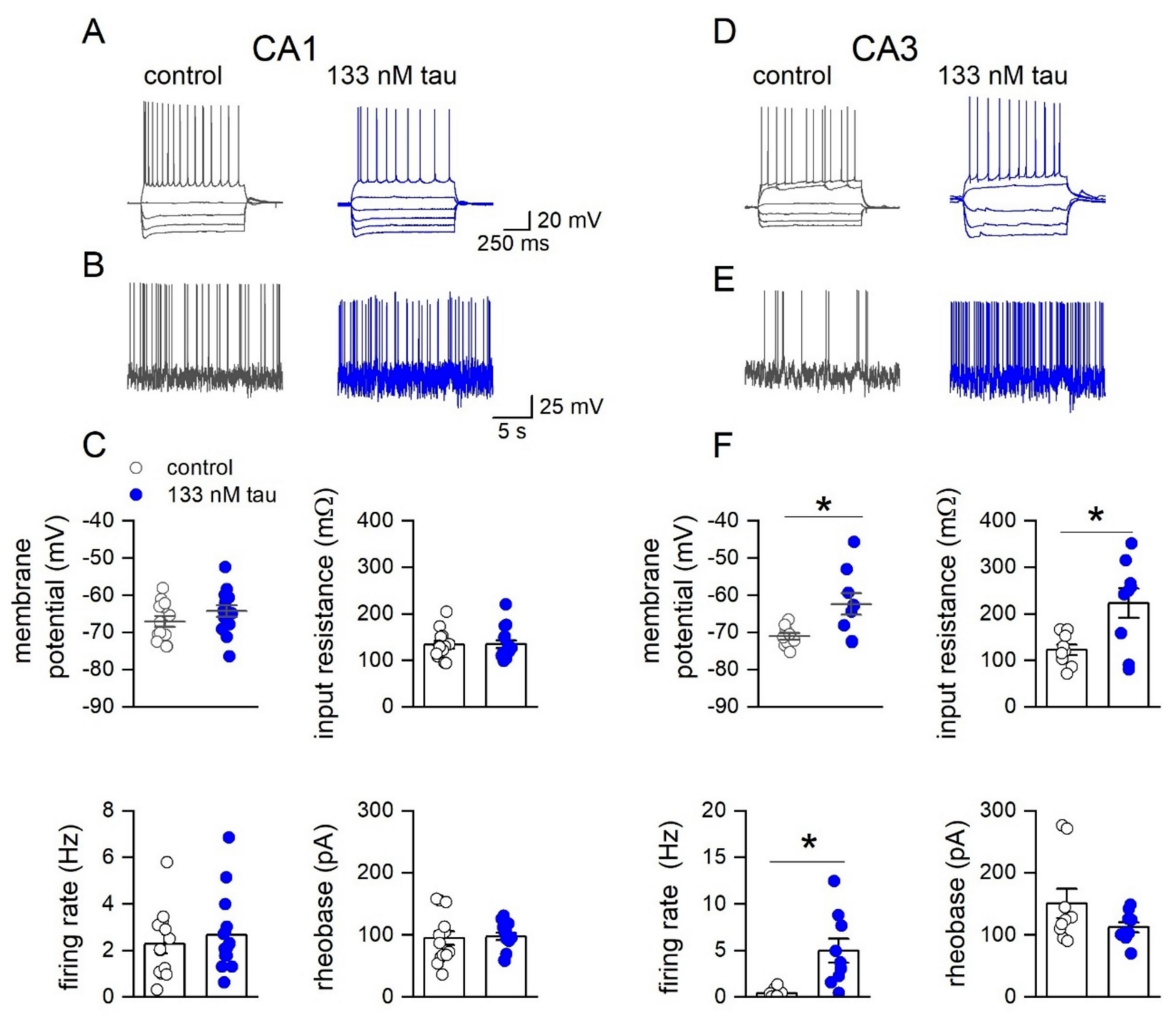
Incubation with soluble recombinant tau aggregates preferentially increases the excitability of CA3 pyramidal cells compared to CA1 pyramidal cells. **(A)** Representative step-current voltage response for a CA1 pyramidal neuron in a control slice and from a slice incubated with tau aggregates (133 nM, 1 h). **(B)** Example voltage responses to naturalistic current injection used to measure firing rate in control and in tau aggregates (see methods). **(C)** Graphs plotting membrane potential, input resistance, firing rate and rheobase for CA1 pyramidal neurons in control slices (*n* = 13) and in slices incubated in tau (*n* = 15). Membrane potential: no significant difference: −69.9 ± 0.8 vs. −68.02 ± 1.4 mV; Mann Whitney U = 63, Z = −1.587 *p* = 0.112. Input resistance: no significant difference: 127.57 ± 9.8 vs. 105.1 ± 10.22 mΩ; Mann Whitney U = 136, Z = 1.72, *p* = 0.084. Firing rate: no significant difference: 2.28 ± 0.65 vs. 2.67 ± 0.45 Hz, Mann Whitney U = 84.5, Z = −0.6, *p* = 0.544. Rheobase no significant difference: 94.58 ± 10.13 vs. 97.6 ± 7.5 pA, Mann Whitney U = 86, Z = −0.51, *p* = 0.652. **(D)** Representative step current–voltage responses for a CA3 pyramidal neuron in a control slice and in a slice incubated with tau aggregates (133 nM). **(E)** Example voltage responses to naturalistic current injection in control and in tau aggregates used to measure firing rate. **(F)** Graphs plotting membrane potential, input resistance, firing rate and rheobase for CA3 pyramidal neurons in control slices (*n* = 9) and in slices incubated in tau (*n* = 9). Membrane potential: There was a significant difference (Mann Whitney, U 14, Z = −2.29, *p* = 0.022), with tau incubated cells depolarised (−71.1 ± 1.2 vs. −62.3 ± 2.5 mV). Input resistance: There was also a significant increase in input resistance (123.2 ± 11.3 vs. 223.4 ± 30.3 mΩ, Mann Whitney, U 17, Z = −2.03, *p* = 0.0423). Firing rate: significant increase in firing rate in tau (0.41 ± 0.2 vs 4.99 ± 1.2 Hz Mann Whitney, U = 52, Z = −2.18, *p* = 0.0289).

Subsequently the effects of extracellular tau aggregates were investigated on the electrophysiological properties of hippocampal pyramidal cells, by incubating hippocampal slices in 133 nM tau aggregates for 1 h. Whole-cell patch-clamp recordings were made from CA1 and CA3 pyramidal cells in control slices (incubated in just aCSF) and in slices that had been incubated in tau aggregates. The tau aggregates had no significant effect on the electrophysiological properties of CA1 pyramidal cells (Figures 3A–C) but did significantly change the properties of CA3 pyramidal cells (Figures 3D–F). These effects (an increase in input resistance, firing rate and a small depolarisation) are consistent with an increase in excitability which could underlie the interictal events.

### Tau aggregates reduce long term potentiation (LTP) in a concentration-dependent manner

**Figure 4:**
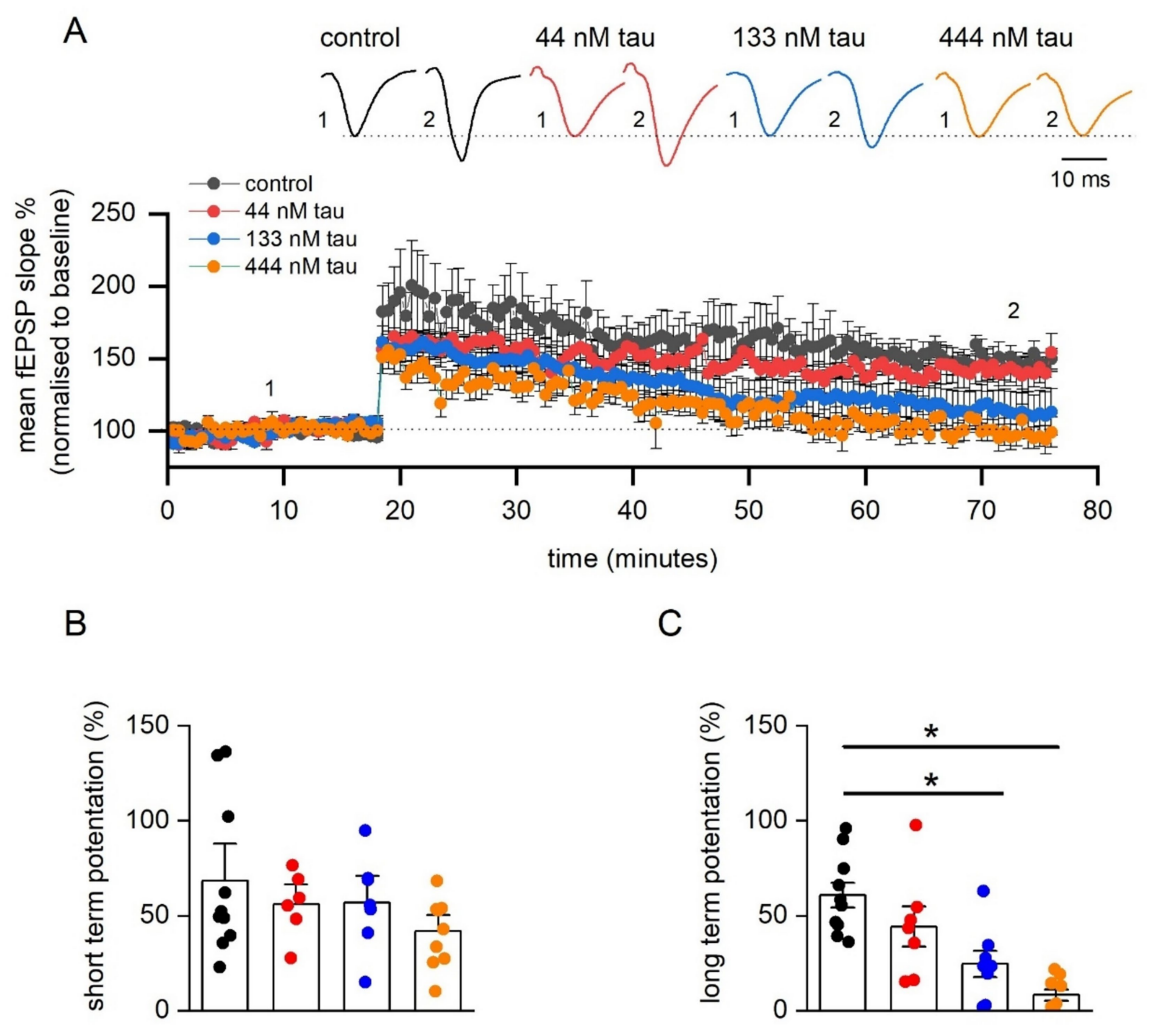
Incubation in soluble recombinant tau aggregates produces a concentration-dependent inhibition of LTP. **(A)** Mean fEPSP slope normalised against baseline plotted against time for control (*n* = 11 slices), 44 nM (*n* = 6 slices), 133 nM (*n* = 7 slices), and 444 tau aggregates (*n* = 9 slices). LTP was induced by high-frequency stimulation (HFS, 100 Hz, 1 s) and recorded for 1 h post LTP induction. Inset, Representative fEPSP waveform averages at the time points indicated by the numerals. **(B)** Graph plotting mean short-term potentiation (STP) of fEPSP slope (measured 3 min post HFS) for the four conditions. A Kruskal-Wallis test demonstrated no significant differences in STP across the groups (H (3) = 1.715, *p* = 0.634). **(C)** Graph plotting mean normalised long-term potentiation of fEPSP slope (measured 55–60 min post HFS) for brain slices incubated in one of the four testing conditions. A Kruskal-Wallis test demonstrated a significant difference in the magnitude of LTP across the four groups (H (3) = 18.77, *p* = 0.0003). Dunn’s test revealed that incubation in either 133 nM tau (*p* = 0.022) or with 444 nM tau (*p* < 0.0001) significantly reduced LTP. Data is presented as mean ± SEM. Data points are values from individual experiments **(B,C)**.

We next investigated the effect of tau aggregate incubation on synaptic plasticity. Following input–output and paired-pulse facilitation recordings, a 20 min baseline was recorded (at ~ 50% maximum) and then LTP was induced with HFS (100 Hz, 1 s) (Figures 4A,C). The degree of LTP was not significantly reduced by 44 nM tau but was by 133 nM tau and 444 nM tau aggregates (LTP abolished in 5 out of 9 slices, Figures 4A,C). Therefore, there is a concentration-dependent effect of tau aggregate incubation on LTP. We also evaluated whether tau aggregates influence short-term potentiation (STP) (Figure 4B, measured as the peak potentiation after LTP induction). None of the concentrations of tau aggregates, had a significant effect on the magnitude of STP. This suggests that the tau aggregates, following a 1 h incubation, can selectively inhibit LTP, without affecting STP.

### Tau aggregates inhibit LTP induction mechanisms rather than just increasing threshold

**Figure 5:**
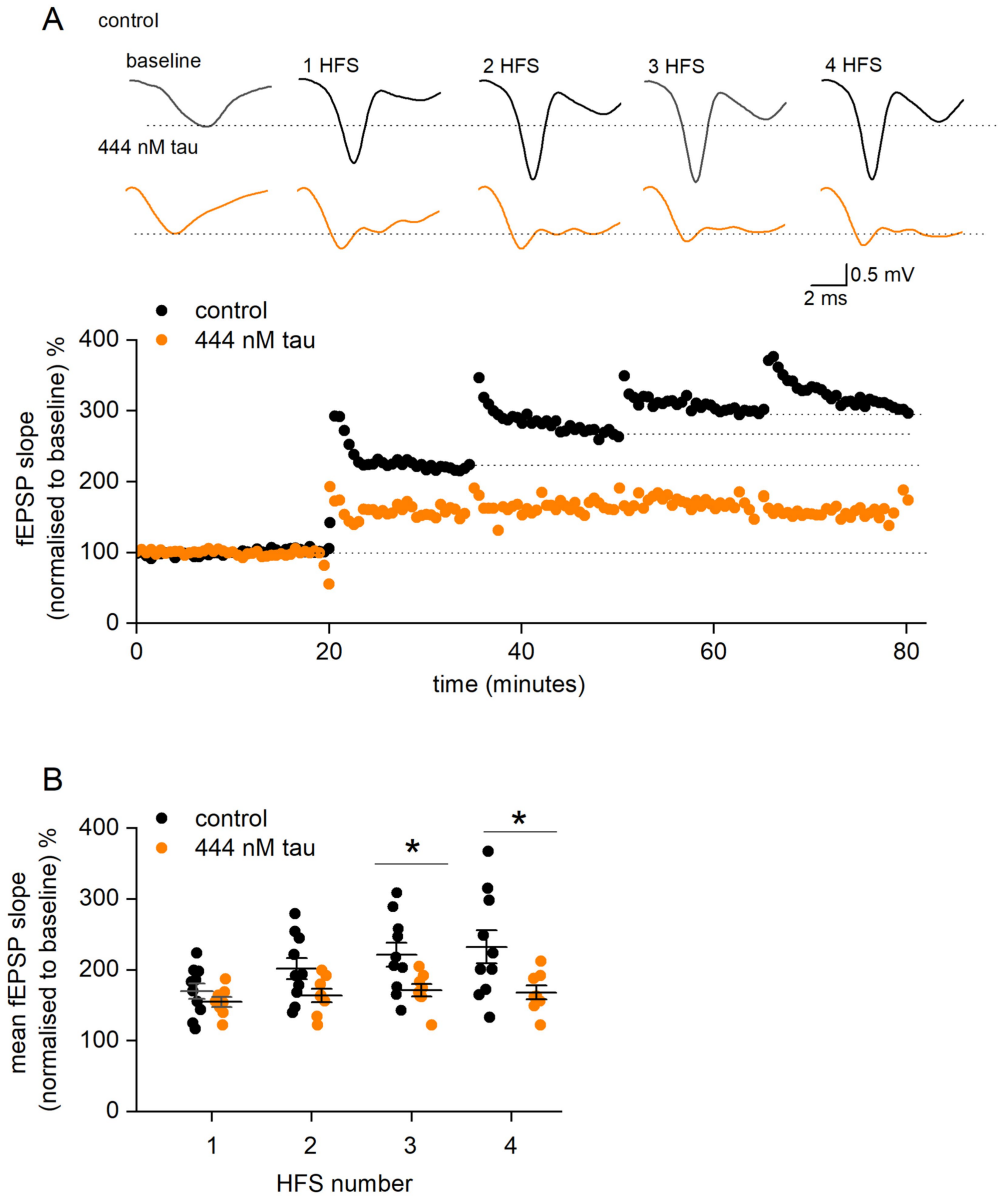
Tau aggregates inhibit LTP induction mechanisms downstream of LTP induction. **(A)** fEPSP slope normalised against baseline plotted against time for a control slice (black circles) and for a slice incubated in 444 nM tau aggregates (orange circles). After a 20-min baseline, a HFS (100 Hz, 1 s) was given every 15 min (numbered 1 to 4). For control, the fEPSP slope increased to about 300% of baseline and then increased no further (saturation). For the slice incubated in tau, there was little increase in fEPSP slope with repeated stimuli. Inset, example average fEPSP waveforms in control and 444 nM tau during baseline after high frequency stimulation (HFS). Note, the repeated stimuli maintain the potentiation of fEPSPs in the tau aggregates, unlike with a single stimulus (Figure 3). **(B)** Graph plotting mean percentage potentiation (and SEM) against stimulus number for control and slices incubated in tau. In control slices, delivery of repeated HFS increased the amount of potentiation from 70.1 ± 10.9% (after a single HFS) to a maximum of 132.5 ± 23.5% (*n* = 10) after 4.1 ± 0.5 stimuli. For slices incubated in tau, the amount of potentiation after one stimulus was 54.8 ± 6.9%, this did not markedly increase after 4 stimuli (59.3 ± 7.8%). The points in (B) are the potentiation values from single experiments. There was not significant difference in potentiation between control and tau after 1 (Mann Whitney U = 27.5, Z = −1.06, *p* = 0.2867) or 2 stimuli (Mann Whitney, U = 20.5, Z = −1.68, *p* = 0.09). However, with 3 or more stimuli there was significantly more potentiation in control vs. tau (for 3 stimuli, Mann Whitney, U = 16, Z = −2.08, *p* = 0.0368 and for 4 stimuli, U = 65, Z = 2.17, *p* = 0.029).

Since incubation of slices with tau aggregates at a concentration of 444 nM either significantly reduced or abolished LTP, a repeated LTP stimulation protocol was used to investigate the mechanism of how tau aggregates inhibit LTP. The rationale for the experiments was that tau aggregates may increase the threshold for LTP induction without inhibiting the downstream mechanisms. If this were the case, it would be predicted that additional stimuli would overcome this inhibition, leading to LTP induction. We used the protocol of Kiyama et al. (1998) and delivered high frequency stimulation (100 Hz, 1 s) every 15 min following LTP induction, until the slope of the fEPSP no longer increased (LTP saturation). In control slices, delivery of repeated HFS significantly increased the amount of potentiation to a maximum after ~4 stimuli (Figures 5A,B). In slices which had been incubated in tau aggregates (444 nM) the amount of potentiation did not markedly increase with repeated stimuli (Figures 5A,B). After the final LTP induction stimulus, in the majority of slices (~ 77%) incubated in tau, the fEPSP slope returned to baseline indicating a lack of LTP. This suggests that the mechanisms for inducing potentiation are inhibited by tau aggregates and it is not a result of simply increasing the threshold for LTP induction.

### Tau aggregates enhance basal synaptic transmission and make it epileptiform in the presence of GABA_A_ receptor antagonists

**Figure 6:**
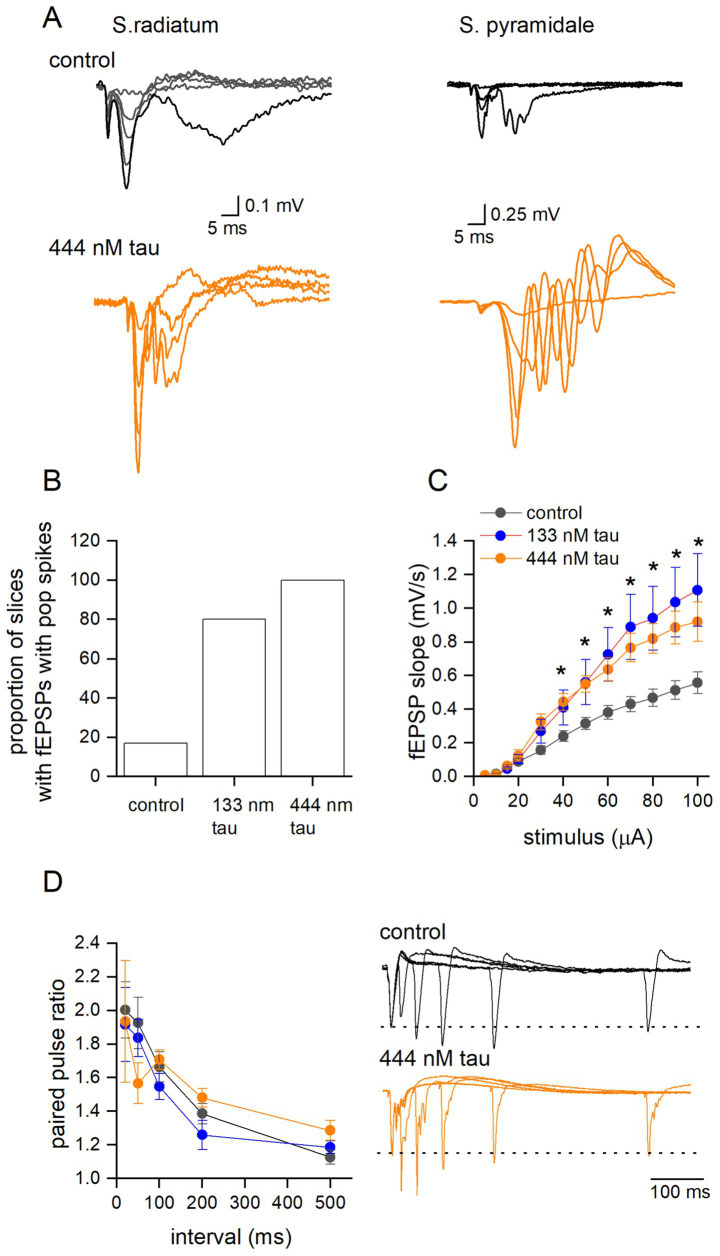
Tau aggregates significantly enhance basal synaptic transmission, but not paired pulse facilitation, in the presence of GABA_A_ and NMDA receptor antagonists. **(A)** Superimposed fEPSP waveforms at increasing stimulus strengths recorded from s. radiatum and s. pyramidale in the same slice which had been either incubated in aCSF (control) or incubated in tau (444 nM). Note the epileptiform pattern of the fEPSP waveform recorded in s. radiatum and the much larger response measured in the s. pyramidale indicative of the increased excitability produced by tau aggregate incubation. **(B)** Graph plotting the percentage of slices that had fEPSPs contaminated with population spikes at baseline (50% of the maximum slope). **(C)** Mean fEPSP slope plotted against stimulus strength for control (black circles) (control aCSF) (*n* = 12 slices), 133 nM (red circles *n* = 10 slices) and 444 nM tau aggregates (orange circles, *n* = 5 slices). There was a significant difference between control and the tau treated slices (Two-way Anova, *F* (2, 288) = 25, *p* < 0.0001). Tukey’s multiple comparison tests confirmed significant differences between control and tau treated slices (significance indicated on figure). **(D)** Mean paired-pulse ratio plotted against paired-pulse interval for control (white circles) (control-aCSF) (*n* = 12 slices), 133 nM tau (*n* = 9 slices) and 444 nM tau aggregates (*n* = 4 slices). There was no significant difference between treatments (Two-way Anova *F* (2, 22) = 0.04, *p* = 0.67). Inset, examples of superimposed average paired pulse traces at intervals 20, 50, 100, 200 and 500 ms for control (top panel) and 444 nM aggregates (bottom panel). All data are represented as mean ± SEM. * indicative of a significant result.

Previous studies have shown that LTP can be inhibited by tau overexpression, but less is known about its effects on group 1 metabotropic glutamate receptor-mediated long-term depression (G1-mGluR-LTD). These forms of synaptic plasticity are mechanistically distinct, and thus the effects of tau aggregates may well be different. To induce G1-mGluR-LTD, slices were perfused with picrotoxin (PTX, 50 μM) and L689,560 (5 μM) to block GABA_A_ and NMDA receptors, respectively. Area CA3 of the hippocampus was removed to reduce the occurrence of spontaneous epileptiform activity which interferes with the synaptic plasticity (Wall et al., 2018; Privitera et al., 2019). Under these conditions, there were clear changes in the waveform of fEPSPs recorded from slices that had been incubated in the tau aggregates (Figure 6A). There was an increase in the occurrence of population spike-contamination of the fEPSPs and burst-like activity following the fEPSP waveform. In control slices, only 2 out of 12 slices (17%) showed population spikes recorded during the baseline (recorded at 40–50% maximum response). In 133 nM tau aggregates, 80% of slices (8 out of 10) were affected and for 444 nM tau, 100% of slices (5 out of 5 slices) had fEPSPs contaminated with population spikes and bursting activity (Figure 6B). Population spikes occur when action potentials are fired at the soma/initial segment leading to an upwards deflection in the fEPSP and are an index for synchronously firing neurons (Andersen et al., 1971; Turner and Richardson, 1991). To investigate this, a recording electrode was placed in stratum pyramidale, and the responses were compared to those when the electrode was placed in stratum radiatum in the same slice. In control slices, only large stimuli evoked action potentials at the soma whereas in slices exposed to tau aggregates not only did the population spikes occur at lower stimulation strengths but were also of a much larger amplitude, presumably indicative of more cells being active (Figure 6A). These effects were not observed under control conditions (see Figure 2), so the simplest explanation is that GABAergic inhibition limits the epileptiform effects of the tau aggregates.

As a result of the change in fEPSP waveform kinetics in the presence of receptor antagonists, we investigated whether tau aggregates also changed basal synaptic transmission under these conditions. We found that tau aggregates significantly enhanced stimulus input–output curves (Figure 6C). Paired pulse facilitation was also examined in the presence of PTX and L689,560 and there were no significant changes, although often the second fEPSP waveform was contaminated with population spikes, in slices that had been incubated in tau aggregates (Figure 6D).

### Tau aggregate incubation increases the magnitude of G1-mGluR-LTD

**Figure 7:**
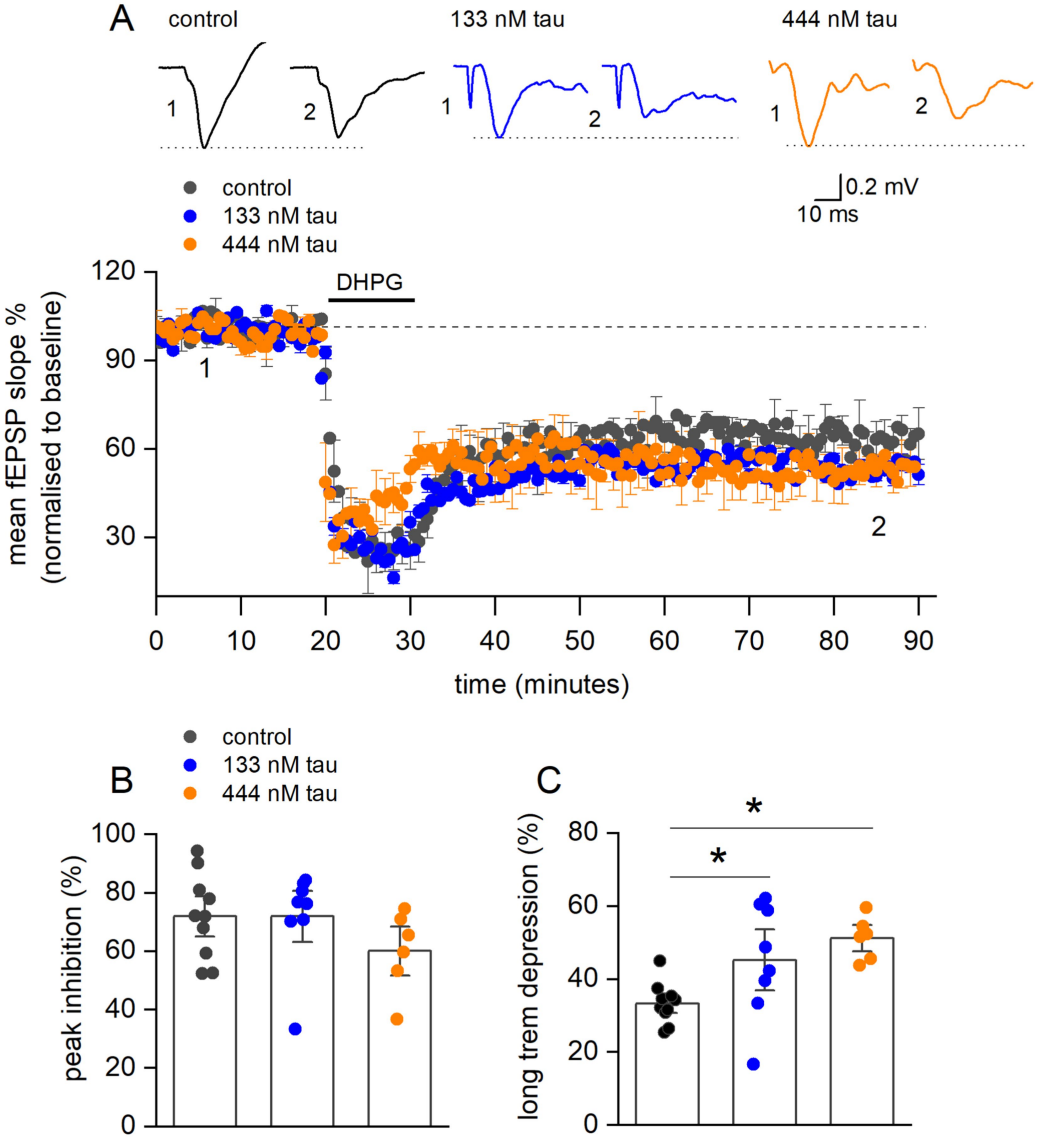
Tau aggregates significantly increase Group 1 metabotropic glutamate receptor mediated long term depression (G1-mGluR-LTD). **(A)** Normalised (to the baseline) fEPSP slope plotted against time for control (black circles) (control-aCSF) (*n* = 7 slices), 133 nM tau aggregates (red circles, *n* = 8 slices) and 444 nM tau aggregates (orange circles, *n* = 5 slices). After a 20 min baseline, mGluR-LTD was induced (see methods). Inset, example average fEPSP waveforms taken from time points denoted by the numerals (1, 2) in control, 133 and 444 nM tau aggregates. **(B)** Bar chart summary of the mean peak inhibition, produced by DHPG. There was no significant change across the conditions (mean inhibition in control 72 ± 3.4%; 133 nM 71.9 ± 7.5% and 444 nM 63.2 ± 7.5% tau aggregates). **(C)** Bar chart summary of mean amplitude of long-term depression (mean depression, control 33.3 ± 1.45, 133 nM 45.26 ± 5.6% and 444 nM 51.21 ± 2.5%). There was a significant increase (Kruskal-Wallis One-way Anova, H(2) = 10, *p* = 0.0065) in the magnitude of depression induced by incubation with 444 nM tau aggregates (Dunns test, *p* = 0.0058) but not with 133 nM tau aggregates (Dunns test *p* = 0.052). In **(B,C)** the data points are values from individual experiments.

To investigate whether tau aggregates modulate G1-mGluR-LTD, fEPSPs were recorded in the presence of PTX and L689,560 in slices with the CA3 region removed. After a 20-min baseline, 100 μM DHPG (a group 1 mGluR agonist) was applied for 10 min to induce LTD. LTD was then recorded for 60 min following DHPG wash (Figure 7A). Although, there was no significant difference in the peak inhibition produced by DHPG (Figure 7B) there was a significant increase in the degree of long-term depression (Figure 7C). These results demonstrate that tau aggregates have differential effects on synaptic weight dynamics by weakening LTP and strengthening LTD.

## Discussion

Because the aggregation of tau into small soluble species occurs in early stages of tauopathies such as Alzheimer’s disease, we have investigated how these aggregates modify hippocampal function, which may contribute to cognitive deficits. We found that tau aggregates enhance circuit excitability and modify synaptic plasticity shifting the dynamic range towards depression.

Our aim was to investigate early changes in hippocampal circuitry, before neuronal degeneration. We have several lines of evidence to support a lack of neural degeneration following the acute 1 h incubation. Firstly, there was no change in the stimulus input output curves in slices incubated in tau aggregates, suggesting no loss of presynaptic or postsynaptic pyramidal neurons (Figure 3A). We also did not observe an increased number of cells with a markedly depolarised membrane potential during whole cell patch clamp recording, which may have been expected if the cells were degenerating.

### Tau aggregates produced from PFFs

In this study we used commercially sourced human tau-441 pre-formed fibrils (PFFs, from rPeptide) to produce soluble tau aggregates (the PFFs were broken down with ultrasonication for 15 min, Polinski, 2018). We have used the term tau aggregates throughout this manuscript as we cannot exclude the possibility that some larger aggregates are still present together with the soluble oligomers following ultrasonication (as size-exclusion chromatography was not used). Using SMMP we found that the aggregates had a mean mass of ~300 kDa which is equivalent to ~ 5–6 monomers. In future studies, techniques such as Western blotting could be used to further characterise the profile of the aggregates as in Wang et al. (2024).

### Incubation of slices in tau aggregates increases circuit excitability

We have previously used an incubation approach to investigate the circuit effects of the tau species present in human (patient-derived) cerebral spinal fluid (CSF, Brown et al., 2023). In that study we developed small bespoke incubation chambers to reduce the amount of CSF required for the experiments. In control experiments, we showed that incubation of slices in these chambers with control aCSF had no significant effects on circuit and neuronal properties (Brown et al., 2023). Here we observed clear effects of recombinant human tau aggregates with a 1-h incubation, strongly suggesting that the tau aggregates are taken up into neurons within this time frame. Studies have shown that exogenous tau species can be internalized by cells *in vitro* (Frost et al., 2009) and by cultured neurons through endocytosis (Wu et al., 2013). In Fá et al. (2016) hippocampal cultures were treated with oTau 4R/2 N to determine whether it could be internalized. Time-lapse confocal imaging demonstrated that oTau 4R/2 N conjugated to IRIS-5 was present in cultured neurons after ~20 min of exposure (Fá et al., 2016). In the future, it would be interesting to investigate different durations of tau incubation, to determine how this changes the observed effects of the tau aggregates.

The tau aggregates used in this study were not phosphorylated. It is possible that during the incubation period the tau aggregates do become phosphorylated, and that this could play a role in their activity. In future studies it would be interesting to use phosphorylated forms of tau aggregates and monomers and compare these to our findings here with non-phosphorylated tau.

One of the key observations in this study, is that incubation with tau aggregates increased the excitability of hippocampal circuits. Incubation with 133 nM of tau aggregates induced interictal events (measured on the extracellular electrode) and preferentially affected CA3 pyramidal cells producing an increase in input resistance and an increase in firing rate. These changes to excitability of the CA3 cells maybe driving the interictal events. It is unclear whether this effect on CA3 pyramidal cells is because they take up the tau aggregates more readily than CA1 pyramidal cells or that they are affected at lower concentrations.

In contrast, when GABA_A_ receptors were blocked, tau aggregates were very pro-epileptic. This was observed as a marked increase in the occurrence of population spikes and burst-like activity following the decay of fEPSPs. Recording in stratum pyramidale revealed much larger responses following tau aggregate incubation, consistent with lower action potential threshold and more active synchronised neurons. Similar increases in circuit excitation were also observed with the tau species present in human CSF (Brown et al., 2023) and it has been reported that epileptic activity occurs in a mouse transgenic line over-expressing human mutant tau, a model of frontotemporal dementia with parkinsonism linked to chromosome 17 (FTDP-17, García-Cabrero et al., 2013). The FTDP-17 model displayed spontaneous epileptic activity and seizures with spike–wave complexes in the EEG, and a higher sensitivity to the GABA_A_ receptor antagonist pentylenetetrazol (PTZ) when compared to age-matched controls (García-Cabrero et al., 2013). These effects of tau on circuit excitability are consistent with the increased occurrence of seizures in AD patients (reviewed in Kamondi et al., 2024).

Interestingly, incubation in tau aggregates had no effect on the input–output curves but did increase paired pulse facilitation. This may reflect changes in calcium dynamics which have been reported for tau oligomers (Decker et al., 2015). This contrasts with the data from Hill et al. (2019), when tau was introduced into the presynaptic neuron it significantly impaired synaptic transmission and increased depression during trains of activity. This may be the result of a smaller amount of tau accumulating within cells when slices were incubated in tau aggregates over a 1 h time period. This could be tested, in future studies, by using longer incubation times. In contrast, blocking of GABA_A_ receptors revealed large changes in stimulus input–output curves. This was not accompanied by consistent changes in PPF and thus maybe be a postsynaptic effect. This data suggest that GABAergic signalling plays an important role in limiting the excitatory effects of tau aggregates.

### Tau aggregates produce a concentration-dependent inhibition of LTP

It is well established that acute application of amyloid oligomers can inhibit LTP (Raymond et al., 2003; Shankar et al., 2008; Wang et al., 2009; Lei et al., 2016) and that this effect requires the presence of endogenous tau protein (Shipton et al., 2011). The evidence for the acute effects of tau aggregates on LTP is less clear (but see Fá et al., 2016; Hill et al., 2019; Ondrejcak et al., 2024). We have previously shown that introduction of tau oligomers into postsynaptic neurons blocks LTP in the hippocampus (Hill et al., 2019). Here we have replicated this effect, with a one-hour incubation with extracellular tau aggregates and found a concentration-dependent decrease in LTP, with almost complete abolition with 444 nM tau aggregates. These effects were only accompanied by changes in short-term potentiation (STP) for 444 nM. When tau was induced directly into the postsynaptic neuron it reduced both STP and LTP at all concentrations (Hill et al., 2019). This may be a time-dependent phenomenon, with insufficient tau entering neurons to block STP. To test this, longer tau aggregate incubations could be used. There is evidence that the mechanisms underlying STP and LTP are different (for example see France et al., 2022) and thus STP maybe more resistant to the effects of tau than LTP.

We examined the mechanism for the inhibition of LTP, by giving repeated LTP induction stimuli every 15 min (based on Kiyama et al., 1998). We found that that the impairment of LTP induction was not attributable solely to an increase in the threshold for LTP induction but was also a consequence of the inhibition of downstream mechanisms. It is well established that in the CA1 region of the hippocampus, the degree of NMDA receptor channel activation determines the threshold for plasticity (Malenka, 1991) and the amount of Ca^2+^ influx through the NMDA channel regulates the magnitude and direction of changes in synaptic efficacy (Perkel et al., 1993; Cummings et al., 1996). When NMDA receptor channels are activated repeatedly (by multiple trains of tetanic stimulation), the resulting LTP is impaired in slices incubated in tau aggregates, thus cannot be attributed solely to a reduction in Ca^2+^ influx but also to effects on LTP expression mechanisms that are downstream from the Ca^2+^ influx. One such mechanism is the trafficking of AMPA receptors to the synaptic membrane. It has been reported that tau interacts with, and dose-dependently reduces, the activity of N-ethylmaleimide sensitive fusion protein (NSF), a vesicular ATPase essential for AMPA-type glutamate receptor (AMPAR) trafficking (Prikas et al., 2022).

### Incubation with tau aggregates enhances group1 metabotropic group 1 long term depression (G1-mGluR-LTD)

We have also demonstrated that incubation of slices with either 133 or 444 nM tau aggregates significantly increased the magnitude of G1-mGluR-LTD. This occurred without a significant change in the amplitude of short-term depression (STD), suggesting no significant changes in mGluR5 expression. Impaired/enhanced mGluR-LTD has been associated with deficits in acquisition/consolidation of spatial learning and poor performance in task reversal (Eales et al., 2014; Mills et al., 2014; Privitera et al., 2019; Wall et al., 2018). G1-mGluR-LTD occurs via a rapid increase in Arc protein expression leading to AMPA receptor internalisation (Waung et al., 2008). Increases in Arc expression and increases in its stability led to greater G1-mGluR-LTD (Wall et al., 2018). In some studies tau has been linked to Arc stability (Yakout et al., 2024) but in others there is no clear link between tau pathology and Arc responses (Rudinskiy et al., 2014).

The inhibition of long-term potentiation coupled with an increase in G1-mGluR-LTD by tau aggregates shifts the synaptic capacity range towards depression. In a mouse model of AD (APP_swe_/PS1dE9) there is a similar shift in synaptic range with an increase in G1-mGluR-LTD and a decrease in LTP at 7 months of age which is associated with significant disruptions in cognitive flexibility (Privitera et al., 2022).

## Significance statement

The aggregation of the protein tau is a key pathological hallmark of neurodegenerative disease such as Alzheimer’s disease (AD). Soluble tau aggregates, formed early in disease, have been shown to alter neuronal function and synaptic plasticity. Here we have investigated the effects of these soluble tau aggregates on acute mouse hippocampal slices to understand early circuit changes. Tau aggregates shift synaptic plasticity towards depression and increase neuronal excitability, effects which could contribute to changes in cognition and development of seizures observed in AD patients.

## Conclusion

Here we have shown that a short incubation with nanomolar concentrations of tau aggregates can induce distinct changes in hippocampal synaptic plasticity shifting the dynamic range of synapses toward depression. Tau aggregates also induce marked increases in network excitability, an effect which is dampened by GABAergic inhibition. This effect is consistent with the increased occurrence of seizure activity observed in AD patients.

## Data Availability

The raw data supporting the conclusions of this article will be made available by the authors, without undue reservation.
